# Supplementary material for: A conserved mechanism drives partition complex assembly on bacterial chromosomes and plasmids
Source: Mol Syst Biol. 2018 Nov 16;14(11):e8516. doi: 10.15252/msb.20188516 (PMC6238139; doi:10.15252/msb.20188516)
Supplement: Supplementary file 1 — Appendix [file MSB-14-e8516-s001.pdf]

Appendix

Conserved mechanism for dynamic partition complexes assembly on bacterial chromosomes and plasmids

Roxanne E. Debaugny, Aurore Sanchez, Jérôme Rech, Delphine Labourdette, Jérôme Dorignac, Frédéric Geniet, John Palmeri, Andrea Parmeggiani, François Boudsocq, Véronique Anton Leberre, Jean-Charles Walter and Jean-Yves Bouet

Appendix table of content

- Appendix Figures:

Appendix Figures S1: $ParB_F$ binding profiles is invariant in forward and reverse orientation of *parS*$_F$ at the *xylE* locus on *E. coli* chromosome, and with $ParB_F$ expressed *in trans*.

Appendix Figure S2: $ParB_F$ DNA binding profile in the vicinity of *parS*$_F$ follow a stochastic probability independently of its intracellular level.

Appendix Figure S3: The $ParB_F$-3R* variant (box II) is deficient in cluster assembly in WT and fluorescent fusion versions.

Appendix Figure S4: $ParB_{Vcho}$ binds slightly differently to the three *parS*$_{Vcho}$ sites.

Appendix Figure S5: Variation in nucleoid compaction in function of growth conditions.

Appendix Figure S6: ParB dynamics between partition complexes, measured by FRAP experiments.

- Appendix Tables:

Appendix Table S1: *E. coli* strains and plasmids

Appendix Table S2: The plasmid F1-10B *parB*$_F$*-mVenus* is fully stable in *E. coli* cells grown in exponential phase but not the mini-F *parB*$_F$*-3R**

- Appendix References

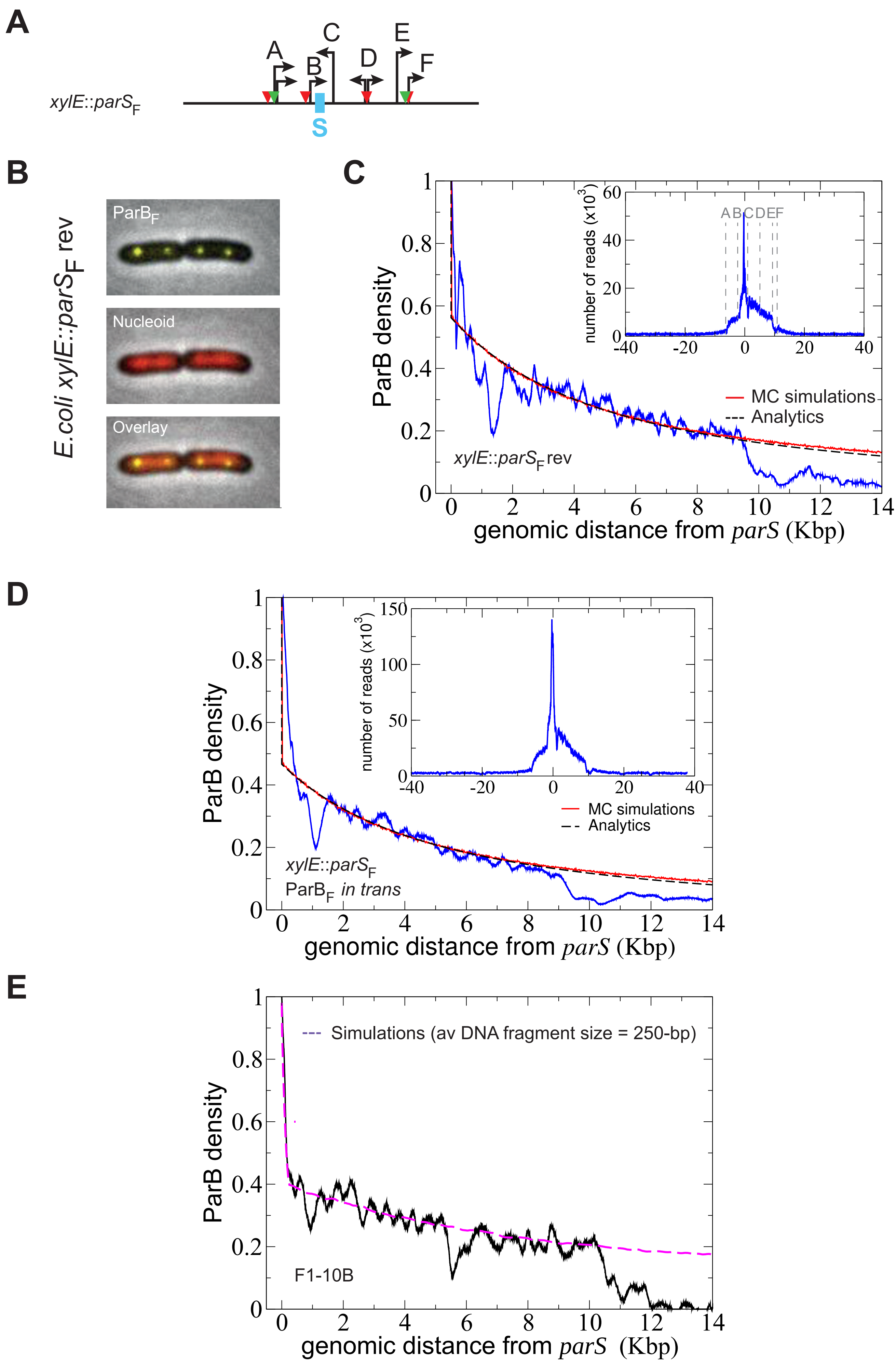

Appendix Figure S1

Appendix Figure S1: ParB_F binding profiles is invariant in forward and reverse orientation of *parS*$_F$ at the *xylE* locus on *E. coli* chromosome, and with ParB_F expressed *in trans*.

(A) Schematic representation of *E. coli* chromosome displaying some characteristics in the vicinity of *parS*$_F$ (blue rectangle) inserted in *xylE* in forward orientation. Green and red triangles represent DNA regions bound by IHF and transcriptional regulators, respectively. Oriented arrows indicate the presence of promoters in front of the following open reading frames. Locus A: *yjbEFGH* operon with 2 RcsAB and 1 IHF regulatory binding sites; B: *psiE* with 2 PhoB and 1 CRP-cAMP regulatory binding sites; C: *xylE*; D: divergently transcribed *malEFG* and *malKLM* operons with a complex regulatory region composed of 4 CRP-cAMP, 5 MalT and 2 Fis DNA-binding regulatory sites; E: *yjbI*; F: *ubiCA* operon with 1 Fnr and 1 IHF regulatory binding sites.

(B) *in vivo* ParB clusters on *parS*$_F$ inserted in *xylE* in reverse orientation (*xylE*::*parS*$_F$-rev). Bright field combined with epifluorescence microscopy of *E. coli* cells (DLT3491) is displayed as in Fig. 1B and 1D. Cells were grown in the presence of 100 µM IPTG and harbored a ParB_F/*parS*$_F$ ratio relative to plasmid F of 10 (measured by Western blot; see legend Appendix Fig. S2B).

(C) ParB_F binding profile from *xylE*::*parS*$_F$-rev is identical to the one from *xylE*::*parS*$_F$. The ChIP-seq profile, obtained from strain DLT2076, is displayed as in Fig. 1E. Monte Carlo simulations and analytic description, represented in red and dotted black lines, respectively, are performed as in Fig. 1E, with κ= 0.57. *Inset*; The ParB_F binding profile is represented as the number of sequencing reads over 80-kbp centered at *parS*.

(D) ParB_F binding profile is invariant with ParB_F expressed *in cis* or *in trans*. The ChIP-seq profile obtained from strain DLT3567 (*xylE*::*parS*$_F$) expressing ParB_F *in trans* (from plasmid pJYB299) is displayed as in Fig. 1E. It shows that the ParB_F DNA binding pattern is highly similar to the one observed when ParB_F is expressed *in cis* (from the chromosome). This data also serves as a replicate of the ChIP-seq from strain DLT2075.

(E) Modeling the ChIP-seq data with the integration of the average fragments size of the DNA library. The same experimental ChIP-seq data as in Fig. 1C, from the plasmid F (F1-10B), were simulated by taking into account the average size of the DNA fragments present in the sequencing library. In Fig.1C, ChIP-Seq data was compared with simulations assuming a base pair resolution detection of ParB. However, ChIP-seq technique is based on the sequencing of sonicated fragments (~250-bp) with an intrinsic unknown on the precise location of ParB. The experimental signal is thus artificially enriched masking the sharp decrease between *parS* sites and non-specific DNA. Here, the chosen convention to build the

ParB profile is to count +1 read at each bp of the fragments on which one bound ParB is detected. From the simulation perspective, this amounts to perform two averages: (i) over the bound-ParB positions and (ii) over all fragment positions. Therefore, the distribution of reads over the fragments when a bound ParB is detected is a triangular profile centered at the ParB position where it takes the value of 1 and decreases linearly down to 0 at a genomic distance +/- the fragment size. This modifies the value of κ (and thus N_t) for the simulation (κ= 0.09) compare to Fig. 1C (κ= 0.42).

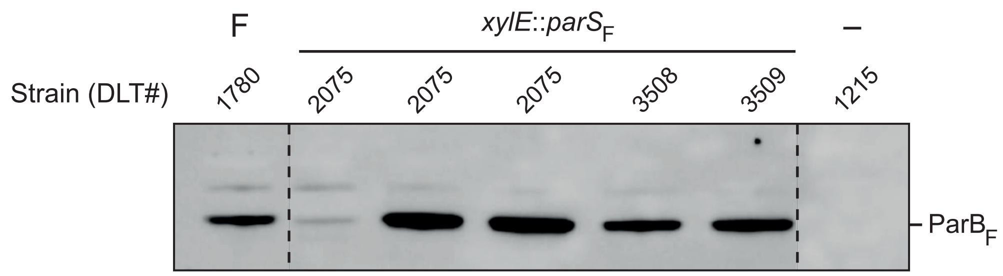

IPTG (µM)	–	0	100	500	100	100	–
Dilution	1	1	5	10	5	5	1
Relative ParB level	1	0.2	8	14	8	8	na
Relative ParB/*parS* ratio	1	0.4	16	28	0.04	0.016	na

Appendix Figure S2: Measurement of the intracellular $\mathrm{ParB_F}$ concentration.

A typical Western blot using anti ParB antibodies is displayed for the indicated strains grown in exponential phase. When indicated, IPTG was added to the cultures to induce $parB_\mathrm{F}$ expression from the *lac* promoter. Quantifications of $\mathrm{ParB_F}$ amount takes into account the dilution of the samples with DLT1215 protein extracts. The $\mathrm{ParB_F}$ level was normalized relatively to strain DLT1780 carrying the WT mini-F expressing $parB_\mathrm{F}$ under its endogenous promoter. The relative ParB/*parS* ratio takes into account the two-fold difference in copy number of the chromosome and the plasmid F, and was estimated from four independent measurements.

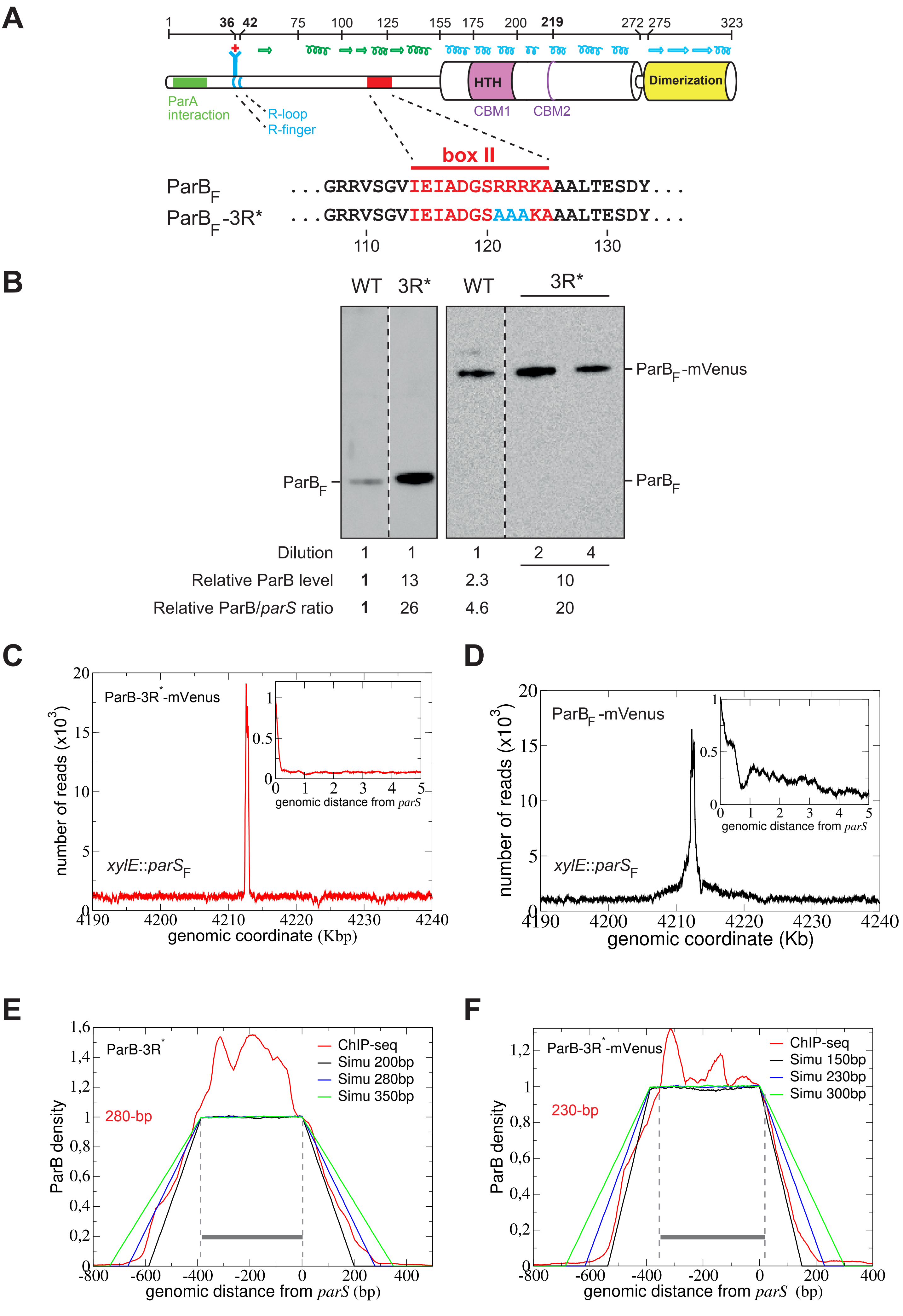

Appendix Figure S3

Appendix Figure S3: The ParB$_F$-3R* variant (box II) is deficient in cluster assembly in WT and fluorescent fusion versions.

(A) Schematic representation of ParB$_F$. *Top*; amino acid numbering and secondary structures of ParB$_F$ to scale, highlighting some characteristic positions. Helices and arrows represent α-helix and β-sheet, respectively, predicted (green) or determined by crystal analysis (blue). *Middle*; functional domains and motifs of ParB$_F$. Thick cylinders (amino acids 155-272 and 275–323) represent the parts of ParB$_F$ structure that have been solved by X-ray crystallography whereas thin cylinders (1-155 and 272-275) have not (Schumacher et al, 2010). Arginine-finger like (R-finger), arginine-loop like (R-loop) and centromere binding motifs (CBM1 and CBM2) were functionally defined previously (Ah-Seng et al, 2009; Sanchez et al, 2013). The box II motif is represented by a red rectangle. *Bottom*; Amino-acids sequences of the box II motif (red) from WT and ParB$_F$ -3R* variants. The three modified arginine (R) residues substituted to alanine (A) are indicated in blue.

(B) Measurement of the intracellular ParB$_F$ level. The quantification of ParB$_F$, ParB$_F$-3R* and ParB$_F$-3R*-mVenus level was performed by Western blot analyses as described in Appendix Fig. S2B from strains DLT3567 (ParB$_F$), DLT3726 (ParB$_F$-3R*), DLT3055 (ParB$_F$-mVenus) and DLT3566 (ParB$_F$-3R*-mVenus). The relative levels of ParB$_F$ and ParB$_F$-mVenus from pDAG114 and pJYB234, respectively, was previously determined at 2.3 (Sanchez et al, 2015). The relative ParB$_F$/*parS*$_F$ ratio (see legend Appendix Fig. S2B) takes into account the two-fold difference in copy number of the chromosome and the plasmid F, and was estimated from two independent measurements.

(C) The DNA binding profile of ParB$_F$-3R*-mVenus is identical to ParB$_F$-3R*. The ChIP-seq profile obtained from strain DLT3566 expressing ParB$_F$-3R*-mVenus fusion protein is displayed as in Fig. 3C. The DNA binding profile of ParB$_F$-3R*, produced in 20-fold excess (see B), indicates that the mVenus tag is not promoting measurable ParB spreading that would arise from enhanced multimerization activity.

(D) The DNA binding profile of ParB$_F$-mVenus is similar to ParB$_F$. The ChIP-seq profile obtained from strain DLT3548 expressing ParB$_F$-mVenus *in trans* from plasmid pJYB294 is displayed as in Fig. 3C. Cells were grown in M9 minimal medium at 30°C.

(E) ChIP-sequencing data modeling accounting for the average size distribution of the DNA fragments. The ParB$_F$-3R* DNA binding profile (red curve) is plotted from ChIP-seq data (same dataset as in Fig. 3C; strain DLT3726) generated from an IP library of DNA fragments of 280-bp in average. Three simulated profiles using a distribution of the size of the DNA fragments of 200-, 280- and 350-bp takes are displayed by black, blue and green curves,

respectively. The grey line represents the position of the 10 specific $ParB_F$ binding sites. The basal readout of ParB binding (background) was subtracted from the data before signal normalization to 1 by the value at the rightmost site of *parS* (genomic coordinate 0).

Here, the modeling describes only the ParB specific DNA binding on *parS* sites (see Materials and Methods). An ideal experiment at the base pair resolution would thus lead to ten discrete peaks of width 16-bp and height 1 at each of the ten *parS*$_F$ sites where a $ParB_F$ is always bound. The simulation using 280-bp well described the decay of experimental data, thus indicating that the $ParB_F$-3R* has lost the capacity to interact with nsDNA in the vicinity of *parS* sites. The discrepancy with the height of the plateau is not explained and could arise from factors not accounted in the simulations.

(F) As in (E) with $ParB_F$-3R*-mVenus. The distribution of the size of the DNA fragments used to generate the IP DNA library (strain DLT3566) has an averaged value of 230-bp. We noticed that the simulations with fragments size of 230-bp reproduced the decay of the profile.

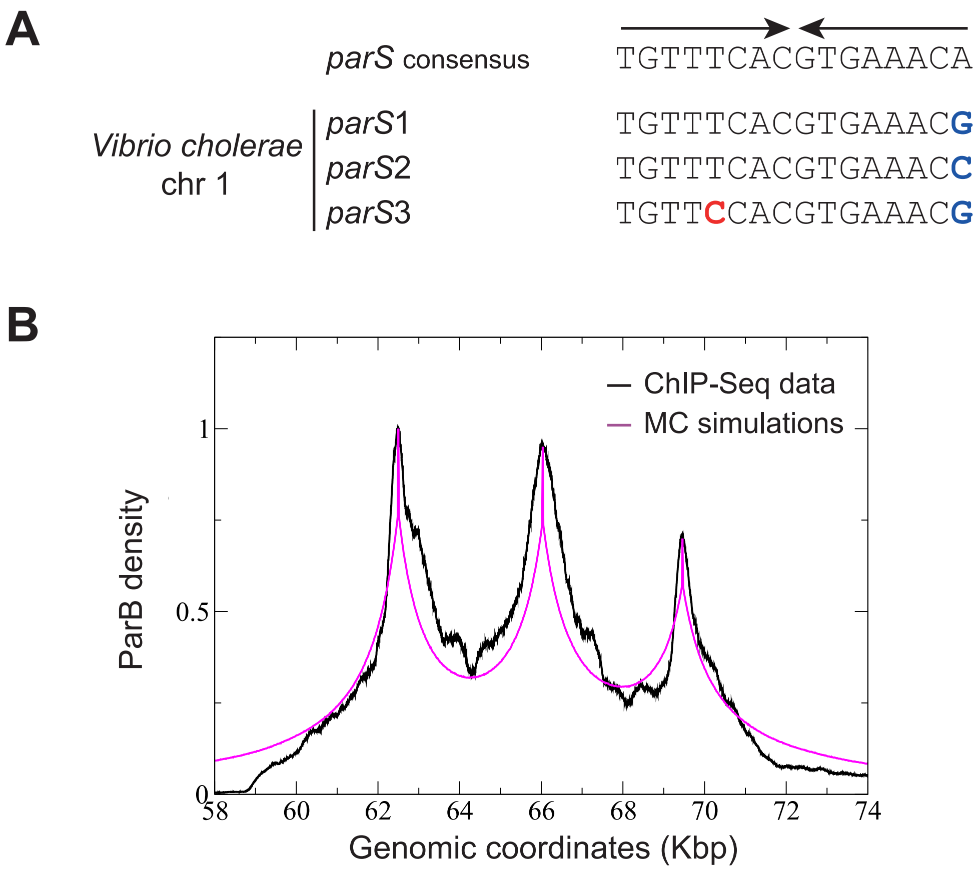

Appendix Figure S4: ParB_{Vcho} binds slightly differently to the three $parS_{Vcho}$ sites.

(A) Nucleotide sequence of the three *parS* sites present on the main chromosome (Chr 1) of *V. cholerae.* The palindromic *parS* sequence indicated on top corresponds to the consensus defined for all *parS* present on the vast majority of bacterial chromosome (Lin & Grossman, 1998). *parS*1 et *parS*2 differ from the consensus at the last position (blue) and *parS*3 at the 5th (red) and last (blue) positions.

(B) Ideal simulation of the ParB_{Vcho} DNA binding profile. The same Chip-seq dataset as in Fig. 4B (modeled with the correction for the distribution of the size of the DNA fragment in the sequencing library; see also Fig. S1E for further explanations) is modeled with ideal simulation (at the bp resolution). The Kuhn length a=16-bp and the foci size σ=25nm remain the same but κ (and thus N_t) was adjusted to κ= 0.47 instead of κ= 0.15 in Fig.4B.

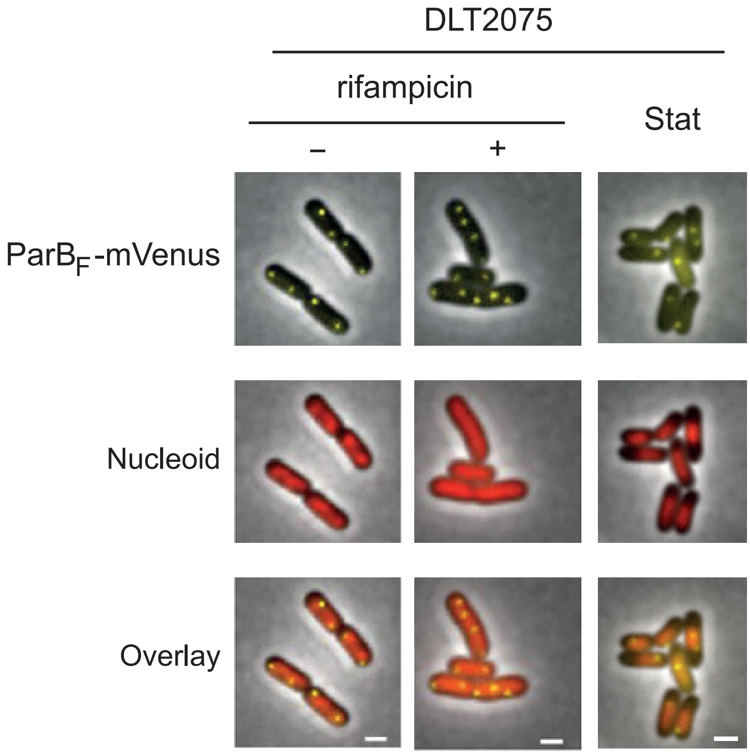

Appendix Figure S5: Nucleoid compaction in different growth conditions.

The strain DLT2075 carrying *xylE*::*parS*$_F$ was grown at 30°C in exponential phase (OD_{600} ~0.6) before addition or not of rifampicin (200 µg.ml^{-1}) and further incubation for 1 hour, or in stationary phase for ~24 hr (Stat). Bright field combined with epifluorescence microscopy displays ParB$_F$-mVenus protein (top), the nucleoid labelled with Hu-mCherry (central) and the overlay of all fluorescent channels (bottom). White bars: 1 µm

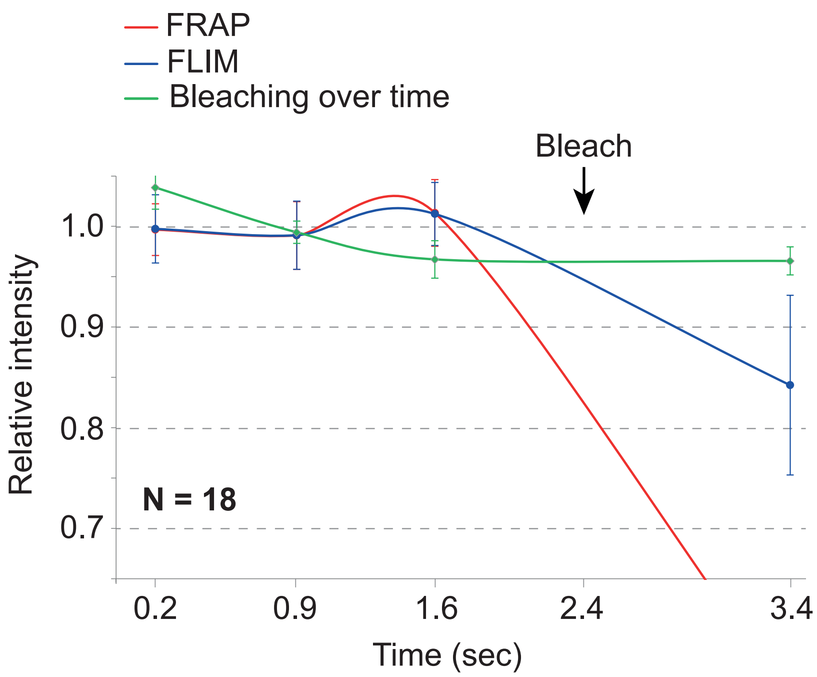

Appendix Figure S6: ParB dynamics between partition complexes, measured by FRAP experiments.

Same as Fig. 6B with zoom in to display the data from 18 independent FRAP experiments between 0 and 4 seconds. The photobleaching with the 488 nm laser was performed on 1 focus on two-foci cells at ~2.4 sec (bleach). For each experiment, the normalization to 1 of the fluorescence intensity was performed by averaging the foci intensity from the three pre-bleached images. Legends are as in Fig. 6B.

Appendix Table S1: Bacterial strains and plasmids

All *Escherichia coli* strains are derivatives of *E. coli* K12, except C2833-based strains deriving from *E. coli* B. The two *Vibrio cholerae* strains used in this study are also listed.

Strains	Genotype/relevant properties	Source/references
C2833	*F', lacIq / lon, lacZ ::T7gene1* (only relevant genotype)	NEBioLabs
C6706	*V. cholerae ,lac, parB1::Tn::kan*	(Cameron et al, 2008)
DLT1215	*F⁻, thi leu thyA deoB supE rpsL, Δ(ara-leu, zac3051::Tn10*	(Bouet et al, 2005)
DLT1471	DLT1215 *lacZ'*:: *PparAB*$_F$ ::*'lacZ*	This work
DLT1472	DLT1215 *lacZ'*:: *parB*$_F$ ::*'lacZ*	(Bouet et al, 2006)
DLT1780	DLT1215 / pDAG114	(Sanchez et al, 2015)
DLT2073	DLT1215 *xylE*::*parS*$_F$	This work
DLT2074	DLT1215 *xylE*::*parS*$_F$ rev	This work
DLT2075	DLT1472 *xylE*::*parS*$_F$	This work
DLT2076	DLT1472 *xylE*::*parS*$_F$ rev	This work
DLT2317	C2833 / pYAS6	This work
DLT2493	C2833 / pYAS25	This work
DLT3053	DLT1215 *Hu-mCherry*, FRT-Kan-FRT	(Le Gall et al, 2016)
DLT3055	DLT3053 / pJYB234	This work
DLT3289	DLT3053 *Hu-mCherry*, FRT (Kan^S)	This work
DLT3431	C2833 / pSM826	This work
DLT3491	DLT2074 *Hu-mCherry*, FRT-Kan-FRT / pJYB294	This work
DLT3493	DLT1215 *aceB*::*parS*$_F$, FRT-Kan-FRT	This work
DLT3495	DLT1471 *aceB*::*parS*$_F$, FRT-Kan-FRT	This work
DLT3508	DLT2075 / pZC302	This work
DLT3509	DLT2075 / pJYB57	This work
DLT3548	DLT2073 / pJYB294	This work
DLT3550	DLT3495 *aceB*::*parS*$_F$, FRT (Kan^S)	This work
DLT3553	DLT3053 / pJYB294	This work
DLT3566	DLT2073 / pJYB296	This work
DLT3567	DLT2073 / pJYB299	This work
DLT3573	DLT2073 *Hu-mCherry*, FRT-Kan-FRT	This work
DLT3574	DLT3566 *Hu-mCherry*, FRT-Kan-FRT	This work
DLT3576	DLT2073 / pJYB57, pJYB294	This work
DLT3577	DLT2073 / pJYB302, pJYB294	This work
DLT3583	DLT3573 / pJYB259	This work
DLT3584	DLT3573 / pJYB294	This work
DLT3586	DLT1215 / F1-10B	This work
DLT3589	DLT1215 / F1-10B-mVenus	This work

DLT3592	DLT3289 / F1-10B ΔAB	This work
DLT3594	DLT3289 / F1-10B-mVenus	This work
DLT3598	DLT3493, FRT (Kan^S)	This work
DLT3605	DLT3598 Hu-mCherry, FRT-Kan-FRT	This work
DLT3607	DLT3053 / pJYB294	This work
DLT3651	DLT2075, Δ(*pgl-yjbE*)::*kan*	This work
DLT3726	DLT2073 / pJYB303	This work
DLT3818	DLT3053 / pJYB322	This work
DLT3831	DLT3592 / pJYB294	This work
N16961	*V. cholerae* , O1 biovar El Tor	(Fogel & Waldor, 2006)
Stellar	F^-, *endA1, supE44, thi-1, recA1* (only relevant genotype)	Clontech (HST08)

Plasmids	Relevant characteristics	Source/references
F1-10	F^+, *lac*$^+$ tn10	CGSC#6451; K603
F1-10B	F1-10, *ccdB*$^-$ *cat*$^+$	This work
F1-10B ΔAB	F1-10B, *parAB*$^-$	This work
F1-10B-BmV	F1-10B, *parB*$_F$-*mVenus*	This work
pAM238	pSC101, *aad*, Plac-*lacZ'*::mcs	(Bouet et al, 1996)
pAS22	pDAG114, *parB*$_F$-R121A-R122A-R123A-*mVenus*	This work
pAS30	pDAG114, *parB*$_F$-R121A-R122A-R123A	This work
pCP20	*ori*^{Ts}, Cam^R, Amp^R, *flp*$^+$	(Datsenko & Wanner, 2000)
pDAG114	mini-F, *repFIA*$^+$, *ccdB*$^-$, *resD*$^+$, *rsfF*$^+$, *cat*$^+$	(Lemonnier et al, 2000)
pDAG209	pDAG114 Δ*parAB*$_F$	(Bouet et al, 2006)
pDK4	*ori*R, Amp^R, FRT-Kan^R-FRT	(Datsenko & Wanner, 2000)
pFC13	pSC101, *repAl*^{Ts}, *rpsL*$^+$, *cat*$^+$	(Cornet et al, 1994)
pJYB50	pFC13, *lacZ*	This work
pJYB52	pJYB50, *lacZ*::*sopOPAB*	This work
pJYB57	pBSKS+, *bla*, *parS*$_F^+$	(Ah-Seng et al, 2009)
pJYB102	pFC13, *xylE*	This work
pJYB103.1	pFC13, *xylE*::*parS*$_F$	This work
pJYB103.2	pFC13, *xylE*::*parS*$_F$-rev	This work
pJYB213	pDAG114, *parB*$_F$-*eGfp*	This work
pJYB234	pDAG114, *parB*$_F$-*mVenus*	(Sanchez et al, 2015)
pJYB259	pYAS47, Plac::*parA parB*$_F$-*mVenus*	This work
pJYB294	pAM238, Plac::*parB*$_F$-*mVenus*	This work
pJYB296	pAM238, Plac::*parB*$_F$-*3R*-mVenus*	This work
pJYB299	pAM238, Plac::*parB*$_F$	This work
pJYB303	pAM238, Plac::*parB*$_F$-*3R**	This work
pSM826	pET28b, *parB*$_{Vc1}$	Gift from Y. Yamaichi
pYAS6	pTYB1, *bla*, pT7::*parB*$_F$-G324- Sce Intein-CBD	(Ah-Seng et al, 2009)
pYAS25	pYAS6, *parB*$_F$-*3R** -G324	This work
pYAS47	pAM238, Plac::*parAB*$_F$	(Ah-Seng et al, 2013)
pZC302	pBR322, *bla*, *parS*$_F^+$	(Bouet et al, 2005)

Appendix Table S2: The plasmid F1-10B *parB*$_F$-*mVenus* is fully stable in *E. coli* cells grown in exponential phase but not the mini-F *parB*$_F$-*3R**

Plasmids [a]	% Loss rate [b]	
	MGC	LB
pDAG114	0.03	0.04
pDAG209	5.3	3.6
F1-10B	0.05	nd
F1-10B-BmV	0.025	nd
pAS30	nd	3.8

[a] All plasmids were introduced in strain DLT1215.
[b] The loss rate per cell per generation is the average of two measurements, except for pAS30 (one measurement). Experiments were performed in MGC at 30 °C or in LB at 37°C. nd; not determined.

Appendix References

Ah-Seng Y, Lopez F, Pasta F, Lane D, Bouet JY (2009) Dual role of DNA in regulating ATP hydrolysis by the SopA partition protein. *J Biol Chem* **284:** 30067-30075

Ah-Seng Y, Rech J, Lane D, Bouet JY (2013) Defining the role of ATP hydrolysis in mitotic segregation of bacterial plasmids. *PLoS genetics* **9:** e1003956

Bouet JY, Bouvier M, Lane D (2006) Concerted action of plasmid maintenance functions: partition complexes create a requirement for dimer resolution. *Mol Microbiol* **62:** 1447-1459

Bouet JY, Campo NJ, Krisch HM, Louarn JM (1996) The effects on *Escherichia coli* of expression of the cloned bacteriophage T4 nucleoid disruption (*ndd*) gene. *Mol Microbiol* **20:** 519-528

Bouet JY, Rech J, Egloff S, Biek DP, Lane D (2005) Probing plasmid partition with centromere-based incompatibility. *Mol Microbiol* **55:** 511-525

Cameron DE, Urbach JM, Mekalanos JJ (2008) A defined transposon mutant library and its use in identifying motility genes in Vibrio cholerae. *Proc Natl Acad Sci U S A* **105:** 8736-8741

Cornet F, Mortier I, Patte J, Louarn JM (1994) Plasmid pSC101 harbors a recombination site, *psi*, which is able to resolve plasmid multimers and to substitute for the analogous chromosomal *Escherichia coli* site *dif*. *J Bacteriol* **176:** 3188-3195

Datsenko KA, Wanner BL (2000) One-step inactivation of chromosomal genes in Escherichia coli K-12 using PCR products. *Proc Natl Acad Sci U S A* **97:** 6640-6645

Fogel MA, Waldor MK (2006) A dynamic, mitotic-like mechanism for bacterial chromosome segregation. *Genes Dev* **20:** 3269-3282

Le Gall A, Cattoni DI, Guilhas B, Mathieu-Demaziere C, Oudjedi L, Fiche JB, Rech J, Abrahamsson S, Murray H, Bouet JY, Nollmann M (2016) Bacterial partition complexes segregate within the volume of the nucleoid. *Nature communications* **7:** 12107

Lemonnier M, Bouet JY, Libante V, Lane D (2000) Disruption of the F plasmid partition complex in vivo by partition protein SopA. *Mol Microbiol* **38:** 493-505.

Lin DCH, Grossman AD (1998) Identification and characterization of a bacterial chromosome partitioning site. *Cell* **92:** 675-685

Sanchez A, Cattoni DI, Walter JC, Rech J, Parmeggiani A, Nollmann M, Bouet JY (2015) Stochastic Self-Assembly of ParB Proteins Builds the Bacterial DNA Segregation Apparatus. *Cell Syst* **1:** 163-173

Sanchez A, Rech J, Gasc C, Bouet JY (2013) Insight into centromere-binding properties of ParB proteins: a secondary binding motif is essential for bacterial genome maintenance. *Nucleic Acids Res* **41:** 3094-3103

Schumacher MA, Piro KM, Xu W (2010) Insight into F plasmid DNA segregation revealed by structures of SopB and SopB-DNA complexes. *Nucleic Acids Res* **38:** 4514-4526
