# Supplementary material for: A conserved mechanism drives partition complex assembly on bacterial chromosomes and plasmids
Source: Mol Syst Biol. 2018 Nov 16;14(11):e8516. doi: 10.15252/msb.20188516 (PMC6238139; doi:10.15252/msb.20188516)
Supplement: Supplementary file 2 — Expanded View Figures PDF [file MSB-14-e8516-s002.pdf]

Expanded View Figures

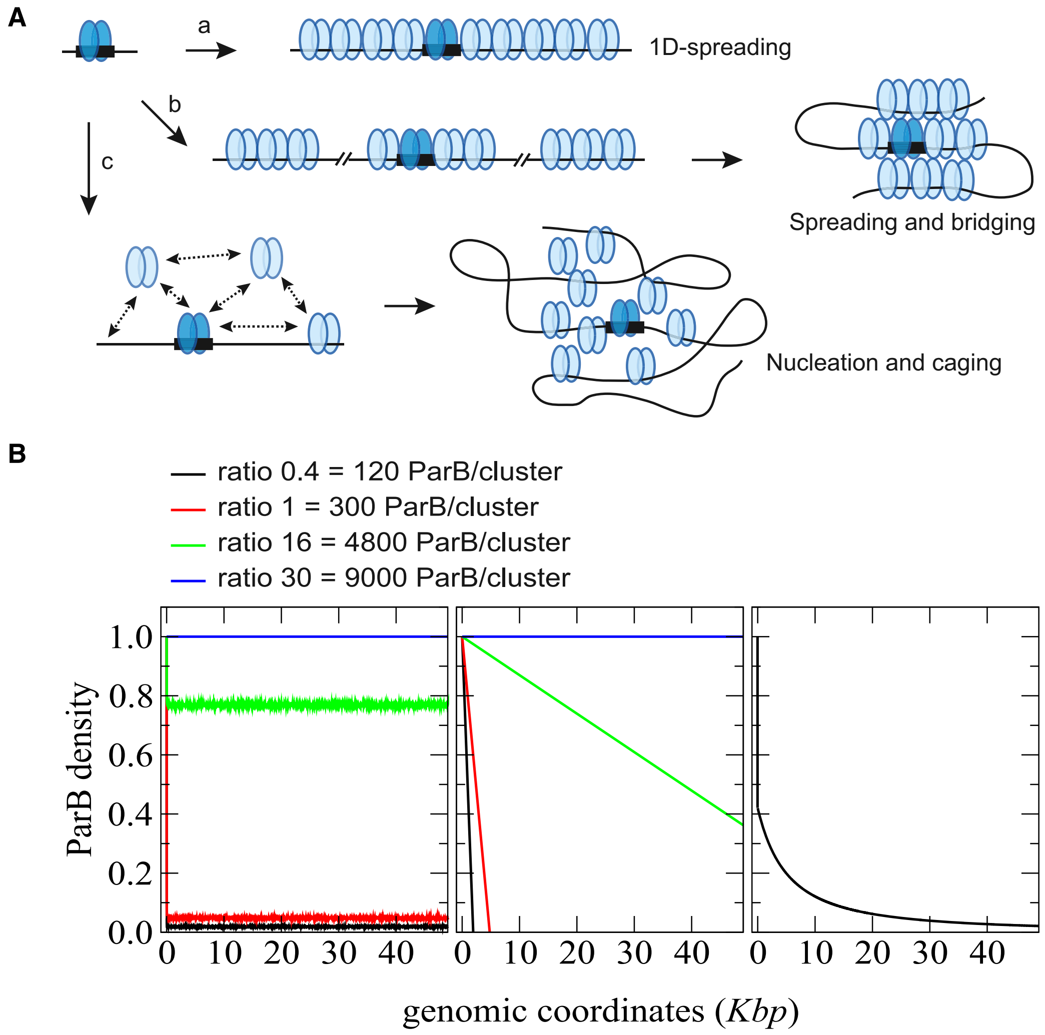

Figure EV1. Physical predictions of the current ParB/*parS* assembly mechanisms.

A Current models for ParB spreading and partition complex assembly. Schematic representation of the main currently proposed mechanisms for the assembly of partition complexes. ParB dimer (dark blue ovoids) binds specifically to the *parS* centromere sequence (black rectangle). (a) "1D spreading". ParB dimers propagate by nearest-neighbor interactions in 1D following the DNA track (black line) and form filaments away from *parS* in both directions. (b) "Spreading and bridging". ParB dimers form (short) 1D filaments on *parS* and on nsDNA by nearest-neighbor interactions. By bridging together these patches of ParB induce the formation of DNA loops. (c) "Nucleation and caging". The transient interactions of ParB with itself and with ParB-nsDNA provide a network of weaker interactions that nucleates the formation of a highly confined ParB zone. By preventing fast ParB diffusion away from the ParB/*parS* complex, these independent but synergistic interactions actively cluster most ParB around *parS*. Importantly, the DNA in the vicinity of parS would preferentially enter this high-density region of ParB. This results in the stochastic binding of ParB over the centromere-proximal DNA sequences which depend on the natural loops of the DNA, and leads to the observed power law decrease in ParB density occurring over large genomic distance.

B Modeling of the evolution of the DNA binding profiles in the vicinity of parS as a function of the ParB level. Schematics of ParB DNA binding profile as a function of ParB concentration from the predictions of the three main physico-mathematical models. Note that the intracellular concentration is a good estimate of the amount of ParB in clusters as over 90% of ParB are highly confined around parS. (Left) The "1D-spreading" model predicts a rapid decrease of the ParB density after the parS site (Broedersz *et al*, 2014). Most of the particles are homogeneously distributed at an average constant value along the DNA. This behavior is explained from general statistical physical ground: a 1D system of particles with nearest-neighbor interactions cannot display a phase transition leading to a global clustering. (Middle) The "Spreading & bridging" model, in the strong coupling limit, predicts a clustering of all ParBs along the DNA with the constraint of overlapping with *parS* site. This leads to a triangular profile with a 1/m slope depending on the number of particles m (Broedersz *et al*, 2014). (Right) With the "Nucleation and caging" model, the decay only depends on the geometry of the foci (discussed in the manuscript). Upon variation of ParB level, the profiles would remain unchanged at a fixed cluster size despite fluctuation in ParB density and would thus overlap after a rescaling of the amplitude. Only "Nucleation and Caging" describes the profiles observed experimentally using high-resolution ChIP-sequencing (Fig 2A).

 © 2018 The Authors
