# Supplementary material for: A conserved mechanism drives partition complex assembly on bacterial chromosomes and plasmids
Source: Mol Syst Biol. 2018 Nov 16;14(11):e8516. doi: 10.15252/msb.20188516 (PMC6238139; doi:10.15252/msb.20188516)
Supplement: Supplementary file 3 — Table EV1 [file MSB-14-e8516-s003.docx]

**Table EV1**: Synoptic of the information relative to the ChIP-sequencing experiments performed in this study.

| Strain  names | Main  properties | Condition  +Induction  IPTG (µM) | ChIP  Input/IP Antibody* | Av.  Library  size** | Reads  total | Reads  mapped | Repli  -cates  *** |
| --- | --- | --- | --- | --- | --- | --- | --- |
| N16961 | *V. cholera*  ParB*_Vc_*_1_ |  | Input  IP antiParB*_Vc_*_1_  Input  IP antiParB*_Vc_*_1_ | 174  242  300  291 | 12,868,709  13,483,693  2,774,742  13,070,887 | 12,858,665  13,455,159  2,604,683  12,667,578 | R1  R1  **R2**  **R2** |
|  |  |  |  |  |  |  |  |
| DLT2075 | *E. coli xylE::parS*  ParB_F_ | 0  100  100  500  100+Rif  100+Rif  100+Rif  100+Rif  100+Stat | IP anti ParB_F_  Input  IP anti ParB_F_  IP anti ParB_F_  Input  IP anti ParB_F_  Input  IP anti ParB_F_  IP anti ParB_F_ | 227  221  232  236  218  220  245  235  221 | 19,383,231  2,749,708  28,121,778  18,640,299  2,574,588  18,713,508  4,729,531  17,497,222  17,421,430 | 18,674,747  2,734,519  27,340,929  18,238,167  2,573,936  18,739,130  4,705,426  17,442,526  16,437,819 | **R1**  **R1**  **R1**  **R1**  R1  R1  **R2**  **R2** **R1** |
|  |  |  |  |  |  |  |  |
| DLT2076 | *E. coli xylE::parS_rev_*  ParB_F_ | 100  100 | Input  IP anti ParB_F_ | 212  205 | 4,677,899  15,913,903 | 4,657,415  15,739,377 | **R1**  **R1** |
|  |  |  |  |  |  |  |  |
| DLT3508 | DLT2075 / pZC302  ParB_F_ | 100  100 | Input  IP anti ParB_F_ | 200  225 | 2,944,026  18,487,262 | 2,932,165  18,280,592 | **R1**  **R1** |
|  |  |  |  |  |  |  |  |
| DLT3509 | DLT2075 / pJYB57  ParB_F_ | 100  100 | Input  IP anti ParB_F_ | 200  207 | 4,971,522  11,633,789 | 4,956,498  11,498,514 | **R1**  **R1** |
|  |  |  |  |  |  |  |  |
| DLT3548 | *E. coli xylE::parS*  ParB_F_-mVenus | 100  100  100-M9^a^  100-M9^a^ | Input  IP anti ParB_F_  Input  IP anti ParB_F_ | 229  223  232  207 | 4,708,965  16,608,073  3,466,564  32,735,443 | 4,694,844  16,495,514  3,451,802  32,610,476 | R1  R1  **R2**  **R2** |
|  |  |  |  |  |  |  |  |
| DLT3566 | *E. coli xylE::parS*  ParB_F_-3R*-mV | 100  100 | Input  IP anti ParB_F_ | 231  217 | 5,692,702  19,676,288 | 5,673,225  19,832,217 | **R1**  **R1** |
|  |  |  |  |  |  |  |  |
| DLT3567 | *E. coli xylE::parS*  ParB_F_ *in trans* | 100  100 | Input  IP anti ParB_F_ | 274  296 | 4,500,397  31,822,212 | 4,479,246  31,385,270 | **R1**  **R1** |
|  |  |  |  |  |  |  |  |
| DLT3586 | *E. coli /* F1-10B  ParB_F_ | -  - | Input  IP anti ParB_F_ | 219  217 | 7,856,173  19,676,288 | 7,772,484  19,480,096 | **R1**  **R1** |
|  |  |  |  |  |  |  |  |
| DLT3651 | *E. coli xylE::parS*  *Δlocus A*  ParB_F_ | 100  100 | Input  IP anti ParB_F_ | 210  196 | 4,858,857  20,060,092 | 4,839,697  19,811,513 | **R1**  **R1** |
|  |  |  |  |  |  |  |  |
| DLT3726 | *E. coli xylE::parS*  ParB_F_-3R* | 100  100 | Input  IP anti ParB_F_ | 303  298 | 2,525,117  15,054,848 | 2,504,280  14,949,822 | **R1**  **R1** |
|  |  |  |  |  |  |  |  |

* Affinity-purified antibodies from rabbit-serum.

** Average size of the DNA fragments in the library as measured by sequencing.

*** Replicates indicated in bold correspond to the ones presented in figures in the manuscript.

^a^ Cells were grown in M9 glucose and casamino-acids at 30°C. All other growth cultures were performed in LB at 37°C.
