# Supplementary material for: A conserved mechanism drives partition complex assembly on bacterial chromosomes and plasmids
Source: Mol Syst Biol. 2018 Nov 16;14(11):e8516. doi: 10.15252/msb.20188516 (PMC6238139; doi:10.15252/msb.20188516)
Supplement: Supplementary file 4 — Table EV2 [file MSB-14-e8516-s004.docx]

**Table EV2:** The patterns of dips and peaks are highly conserved

Strain #1 Growth Strain #2 Growth Correlation Fig.

condition condition coefficient

DLT2075 no IPTG DLT2075 100µM IPTG 0.959 2A

DLT2075 no IPTG DLT2075 500µM IPTG 0.977 2A

DLT2075 100µM IPTG DLT2075 500µM IPTG 0.975 2A

DLT2075 100µM IPTG DLT3651^a^ 100µM IPTG 0.975 5D

DLT2075 500µM IPTG DLT2075 +Rif 0.978 5B

DLT2075 500µM IPTG DLT2075 Stat phase 0.971 5B

DLT2075 100µM IPTG DLT3508 100µM IPTG 0.947 2A

DLT2075 100µM IPTG DLT3509 100µM IPTG 0.935 2A

DLT2075 100µM IPTG DLT3586^b^ 0.814 1C

DLT2075 100µM IPTG Theoretical curve^c^ 0.811 1E

^a^ the strain DLT3651 carries the deletion of the locus A.

^b^ the strain DLT3586 carries the F1-10B plasmid.

^c^ the theoretical curve correspond to the fitted power law in Fig. 1E.

The ParB_F_ DNA binding patterns on the right side of *parS*, from coordinates +222 to + 9057, were compared by correlation analyses. The ChIP-seq pattern from strain DLT2075 (*xylE*::*parS*_F_) grown in the indicated condition (strain #1) is compared with that of the same strain grown in a different condition, of another strain or of a theoretical curve (strain #2). The ParB_F_ DNA binding patterns arising from *xylE*::*parS*_F_ are highly correlated (0.96-0.98) in all tested conditions. Despite the overall shape of the pattern is conserved, these correlation coefficients are higher than the ones comparing *xylE*::*parS*_F_ to F plasmid *parS*_F_ (0.81) or to the theoretical power law distribution (0.81). Also, in the condition of strong ParB titration (strains DLT3508 and DLT3509) which lead to very low ChIP-seq signal in the vicinity of *xylE*::*parS*_F_, the correlation coefficients are still higher (0.93, 0.95) than from the F plasmid *parS*_F_. Therefore, this indicates that the observed dips and peaks are related to the local genomic environment.
